# Supplementary material for: Associations between self-rated health and depressive symptoms among middle-aged and older adults in China: A cross-lagged panel analysis (2011–2020)
Source: PLoS One. 2025 Apr 29;20(4):e0321272. doi: 10.1371/journal.pone.0321272 (PMC12040171; doi:10.1371/journal.pone.0321272)
Supplement: Attachment — Submitted filename: Response to Reviewers.docx [file pone.0321272.s002.docx]

Dear Editor,

We would like to sincerely thank you and the reviewers for taking the time to review our manuscript titled " Associations between self-rated health and depressive symptoms among middle-aged and older adults: A cross-lagged panel analysis（2011-2020）" (manuscript ID: PONE-D-24-54916). We greatly appreciate the constructive feedback provided, which has helped us improve the quality of our work. Below, we have addressed each of the reviewers' comments in detail.

Journal Requirements:

1.Please ensure that your manuscript meets PLOS ONE's style requirements, including those for file naming.

**Response**: We have carefully reviewed the manuscript and ensured that it now meets all the formatting and file naming guidelines as outlined in the journal's instructions for authors.

2.Please note that your Data Availability Statement is currently missing a direct link to access each database.

**Response**: Thank you for your valuable feedback. We apologize for the oversight in the Data Availability Statement. In the revised manuscript, we have now included the direct link to access each database(https://charls.charlsdata.com/pages/data/111/zh-cn.html). Please refer to the updated Data Availability Statement for the complete information.

Reviewer #1:

1. **Comment:** It may be beneficial to specify the geographical context in the title. For example, adding "in China" would provide clarity and relevance.

**Response**: We appreciate this suggestion. In response, we have revised the title to better articulate the geographical context.

2. **Comment:** References (Lines 61–67): The statements made in these lines should be supported by appropriate references. Please cite relevant literature to substantiate your claims.
**Response**: Thank you for your helpful comment. In response, we have added the following references to substantiate our claims in the relevant sections. Please see the revised manuscript for the updated citations.

3. **Comment:** Sample Selection: I am slightly concerned about the sample selection process. It appears that the analysis only included participants who were present in all five waves of data collection. Could the authors clarify whether this approach might introduce bias and how it affects the representativeness of the sample?

**Response**: Thank you for your insightful comment regarding the sample selection. We understand your concern about the potential bias introduced by including only participants who were present in all five waves of data collection. To address this, we would like to clarify that our approach is consistent with established practices in the existing literature. Many studies with similar longitudinal designs have used the same approach of including only participants who completed all waves of data collection in order to ensure data consistency and to reduce potential biases from incomplete data. For example, studies by Chen, Zhang, Duan, Mo, Liu, Ma (1), Ding, Chen and Zhang (2), Liu, Qiao, Wu, Shen and Ke (3), Li, Long, Wu, Zhu, Liu, Zhang (4), Liu, Chen, He, Chen, Liang, Jia (5), Sang, Liu, Zhang, Lu, Wu, Zhang (6)and Zhang, Jia, Li, Li and Yang (7) have followed a similar methodology.

We also attempted to include participants who completed 2, 3, and 4 surveys, but the issue that arose was a large number of missing values. The table below provides the missing data percentages for each variable.

| variable | Gender | Retirement | Residence | age | marital status | per capita household expenditure | Education level |
| --- | --- | --- | --- | --- | --- | --- | --- |
| missing percentage | 24.01 | 24.75 | 24.01 | 24.08 | 24.02 | 41.82 | 24.05 |

| variable | Depressive symptoms | SRH | Financial Support | Daily care | Emotion support | smoking | Health insurance |
| --- | --- | --- | --- | --- | --- | --- | --- |
| missing percentage | 24.01 | 24.01 | 26.61 | 27.99 | 34.76 | 27 | 26.35 |

| variable | drinking alcohol | chronic diseases | ADL | IADL | Pension insurance | social participation |
| --- | --- | --- | --- | --- | --- | --- |
| missing percentage | 24.04 | 26.32 | 24.90 | 24.02 | 32.99 | 24.03 |

We sincerely apologize for not being able to address this issue. We acknowledge its importance and plan to address it in future studies by collecting more comprehensive data and employing appropriate methods.

4. **Comment:** 1) Methodological Considerations: Social Support: As social support is a well-known factor influencing both depressive symptoms and self-rated health, it would be prudent to include it. Economic Status: Economic conditions are another important determinant of both mental and physical health. Please consider including economic status in the analysis or discussing its potential influence on the findings. 2) Correlation Coefficients: The manuscript should specify the types of correlation coefficients used (e.g., Pearson, Spearman) to ensure clarity and reproducibility.

**Response**: 1) Thank you for your valuable suggestion. We fully agree that social support and economic status have a significant impact on depressive symptoms and self-rated health. We have included social support and economic status as covariates in the revised version. Additionally, we have added a discussion on social support and economic status in the methods section. Below, I will provide a detailed explanation of how social support and economic status were measured.

CHARLS does not provide a ready-made measure or scale for social support, and existing literature has not yet reached a consensus on how to measure it. Some studies classify social support into formal and informal types, where formal support is provided by the government and society, while informal support comes from family or friends[8, 9]. Other studies combine indicators such as caregiving time from family members and elderly social participation to measure social support, while some categorize social support into social participation and intergenerational support[10, 11]. In line with the approach adopted by most studies, we divide social support into formal and informal types. Formal support, provided by the government, includes basic medical insurance and pension insurance. The database includes specific questions related to these aspects, such as " Are you the policy holder/primary beneficiary of any of the types of health insurance listed below?" and " Do you currently receive, expect to receive, or contribute to the pension for public servants, or pension for public institution employees, or basic pension for enterprise employees?" A response of "Yes" is coded as 1, and "No" as 0. Informal support, provided by children, friends, and relatives, includes intergenerational support and social participation.

Intergenerational support includes three aspects: emotional support(regular meetings or contacts between participants and their children who do not live together), financial support(financial assistance received by participants from their children in the past year), and caregiving(Whether living with children)[12-14].

Social participation is measured by the question, "Have you done any of these activities in the last month?". The survey encompassed a range of activities, including: (I) Interacting with friends, (II) Playing Mahjong, chess, cards, or visiting a community club, (III) Providing help to someone who does not live with you, (IV) Exercising, (V) Taking part in a community related organization, (VI) Performing voluntary or charity work, (VII) Caring for a sick or disabled adult who does not live with you, (VIII) Attending an educational or training course, (IX) Stock investment, (X) Using the Internet, (XI) Other, (XII) None of these. If participants did not select the (XII) option, they were further asked about the frequency of each social activity on a Likert scale ranging from 0 (No) to 3 (Almost daily), with intermediate options of 1 (Not regularly) and 2 (Almost every week). In 2020, when participants were asked about their social activities in the past month, CHARLS questionnaire removed the previous (IX) and (X) items and combined the (VI) and (VII) items into a new item: “Done voluntary or charity work or cared for a sick or disabled adult who does not live with you.” Apart from this change, all other items remained consistent with those in 2011,2013,2015, and 2018. To maintain consistency in scoring fluctuations across the five waves of data as much as possible, we followed Li, Long (4) approach and incorporated the previously existing (IX) and (X) options from 2011,2013,2015 and 2018 into the (XI) category, labeled ‘Other’. The scores for options (VI) and (VII) were combined, resulting a maximum score of 3 for this combined category. The overall social participation score ranged from 0 to 24, with a higher score indicating a higher level of social participation.

Per capita household expenditure as a measure of economic status. The reasons why we used per capita household expenditure instead of income to represent the participants' economic status are mainly as follows: 1) Measuring income raises many well-known difficulties. Income has much measurement error and varies greatly over years and over the life-cycle. Expenditure is a better measure of long-run resources than is current income, particularly so in low-income rural settings, where incomes can vary so much year to year because of variation in weather, pests, plant diseases, and so on. 2) Expenditure varies much less over time than does income because households try to smooth their consumption. 3)Expenditure includes the value of food production which is self-consumed, which ought to be included in income, but may not be in all measures of income. 4)Expenditure also tends to be measured with less error than income[15, 16].

2) We agree that specifying the type of correlation coefficient is important for clarity and reproducibility. In response to your comment, we have added the relevant details to the manuscript. Specifically, we have clarified that we used the Spearman correlation coefficient in the Statistical analyses, page 11, lines 212.

5. **Comment:** Table (M, SD, Mis): When reporting descriptive statistics such as M and SD, please clarify whether the data are presented as mean and standard deviation, or if a median and interquartile range might be more appropriate given the data distribution.

**Response**: Thank you very much for your thoughtful and constructive comment. Upon reviewing your suggestion, we realized that we had overlooked an important issue regarding the selection of descriptive statistics. Your recommendation to consider the data distribution and to clarify median and interquartile range, has been extremely valuable. As a result, we re-examined the data distribution and presented the skewed data (such as depressive symptoms, age, social participation, per capita household expenditure, and economic support from children) using the median and interquartile range, and we will make this distinction clearer in the revised table caption and the corresponding methods section.

6. **Comment:** Interpretation of Findings (Lines Discussing "Effect"): The manuscript states that the study supports a "negative predictive effect" of SRH on depressive symptoms. However, as this is not an interventional study, it may be more accurate to use terms such as "association" or "relationship" rather than "effect," which implies causality.

**Response**: Thank you for your valuable comment. We acknowledge that the term "effect" may imply causality, which is not appropriate in a non-interventional study. In response to your suggestion, we have revised the manuscript to replace "negative predictive effect" with "negative relationship", which more accurately reflects the nature of the findings.

Reviewer #2:

1.**Comment:** The authors may be more careful when using causal inference. In line 69 and line 70, the authors state that the longitudinal design strengthens causal inference. However, there are three limitations to establish causality. Firstly, there might be unobserved factors such as personal traits and caregiving decision that may simultaneously influence both SRH and depressive symptoms. Secondly, both SRH and depressive symptoms are self-reported, potentially leading to inaccuracies. Finally, the authors do not test for stationarity (I mention it in my second point below) of variables. If the stationary assumption is violated, the causal relationship may no longer hold. Thus, it would be better to replace stronger causal inference with stronger correlation.

**Response**: Thank you for your constructive comments. we have revised the manuscript to replace the phrase "strengthens causal inference" with "stronger correlation" to reflect a more cautious and accurate interpretation of our findings.

2.**Comment:** The authors may find it useful to conduct a stationary test to ensure the relationship between variables remains constant over time. If the data is non-stationary, appropriate transformations or differencing may be required to avoid spurious regression between SRH and depressive symptoms. For instance, changes in SRH or depressive symptoms resulting from structural shifts, such as population aging or the COVID-19 pandemic, cannot conclusively establish a causal relationship.

**Response**: Thank you for your valuable suggestion regarding the stationarity test. We have carefully considered your comment and conducted stationarity tests for both SRH and depressive symptoms to ensure the validity of our analysis. The results of the stationarity tests indicate that both SRH and depressive symptoms are stationary over time. These findings suggest that the relationship between these variables remains stable and is not influenced by non-stationary trends. We have updated the manuscript to include these test results and described the methods used for the stationarity analysis in the method section.

3.**Comment:** The authors might consider adding more time-variant covariates such as employment status or income. The dataset includes individuals aged 45 and older, many of whom may have retired during the survey period. Employment status (or income) could affect SRH and depressive symptoms for two reasons. On one hand, retirement may lead to feeling of upset as individuals may find it difficult to recognize their value as they are no longer needed due to transition out of professional roles. On the other hand, Reduced income after retirement may result in financial pressures, particularly for individuals who still have to support their children or grandchildren.

**Response**: Thank you for your thoughtful suggestions regarding the inclusion of time-variant covariates such as employment status and income. We appreciate your detailed explanation of how these variables could influence self-rated health (SRH) and depressive symptoms, particularly in the context of retirement. In response to your comments, we have added employment status and per capita household expenditure as time-variant covariates in our analysis. The section2.2.3 has been updated to explain how these variables were defined and incorporated into the analysis. The reasons why we used per capita household expenditure instead of income to represent the participants' economic status are mainly as follows: 1) Measuring income raises many well-known difficulties. Income has much measurement error and varies greatly over years and over the life-cycle. Expenditure is a better measure of long-run resources than is current income, particularly so in low-income rural settings, where incomes can vary so much year to year because of variation in weather, pests, plant diseases, and so on. 2) Expenditure varies much less over time than does income because households try to smooth their consumption. 3)Expenditure includes the value of food production which is self-consumed, which ought to be included in income, but may not be in all measures of income. 4)Expenditure also tends to be measured with less error than income[15, 16].

4.**Comment:** It is better if the authors could be clearer about the variables used and reported. In Line 113-115, the authors categorized the CESD-10 score into a binary variable indicating whether have depressive symptoms or not, but fail to provide descriptive statistics of the binary variable indicating the depressive symptoms. It would be better and clearer to provide descriptive statistics on both depressive symptoms and CESD-10 score. Additionally, the authors should specify which variable (CESD-10 scores or depressive symptoms) is used in the statistical analyses presented in Sections 3.2 and 3.3.

**Response**: Thank you for your insightful comments. We have carefully addressed your concerns and made the necessary revisions to the manuscript as follows:1) descriptive Statistics for the Binary Variable Indicating Depressive Symptoms: we have now included descriptive statistics for the binary variable indicating the presence of depressive symptoms. This information is provided in Table 2, ensuring a clearer and more comprehensive understanding of the data.2) Clarification of Variables Used in Statistical Analyses: we illustrated in Section 2.2.1 that cesd-10 continuous scores are used for data analysis in Sections 3.1, 3.2 and 3.3.

5.**Comment:** In the Section 3.2, the authors could be clearer about the expression. For Table 2 in the Section 3.2, it would be better to explain more about the numbers in the first rows. If my understanding is correct, the numbers in the first row aligns with those in the first column, and it would be better to make it clearer in the notes below. In addition, in the Section 3.2, the authors could provide more interpretations about the correlation results so that it would be easier to understand. For instance, the negative relationship between SRH and depressive symptoms indicate individuals with higher.

**Response**: Thank you for your valuable feedback. You are correct that the numbers in the first row align with those in the first column. To make this clearer, we have reviewed and revised Table 2(Table 2 in the revised manuscript has been renumbered as Table 3) and added explicit notes below it. Additionally, based on your suggestion, we have provided a more detailed explanation of the correlation results in Section 3.2(Section 3.2 in the revised manuscript has been renumbered as Section 3.3) as follows: “the negative relationship between SRH and depressive symptoms indicate individuals with higher SRH tend to have lower depressive symptoms; conversely, individuals with lower SRH may experience higher depressive symptoms.”

6.**Comment:** The authors could consider exploring potential heterogeneous effect. For instance, the path coefficients from SRH to depressive symptoms might vary across age groups, gender, or geographic regions. It may help to enrich the study.

**Response**: Thank you for the constructive suggestion. In response, we conducted additional subgroup analyses to explore the potential heterogeneous effects. We examined whether the bidirectional relationship between self-rated health (SRH) and depressive symptoms varies between urban and rural areas. We have updated the manuscript to include multi-group analyses results and added corresponding content to the Abstract, Introduction, Methods, Results, and Discussion sections.

1.	Chen Z, Zhang X, Duan Y, Mo T, Liu W, Ma Y, et al. The Relationship Between Sleep Duration and Blood Lipids Among Chinese Middle-Aged and Older Adults: Cross-Lagged Path Analysis From CHARLS. Frontiers in public health. 2022;10:868059.<https://doi.org/10.3389/fpubh.2022.868059>.

2.	Ding Y, Chen L, Zhang Z. The relationship between social participation and depressive symptoms among Chinese middle-aged and older adults: A cross-lagged panel analysis. Frontiers in public health. 2022;10.<https://doi.org/10.3389/fpubh.2022.996606>.

3.	Liu S, Qiao Y, Wu Y, Shen Y, Ke C. The longitudinal relation between depressive symptoms and change in self-rated health: A nationwide cohort study. J Psychiatr Res. 2021;136:217-223.<https://doi.org/10.1016/j.jpsychires.2021.02.039>.

4.	Li C, Long C, Wu H, Zhu G, Liu D, Zhang C, et al. The impact of internet device diversity on depressive symptoms among middle-aged and older adults in China: A cross-lagged model of social participation as the mediating role. Journal of Affective Disorders. 2025;368:645-654.<https://doi.org/10.1016/j.jad.2024.09.037>.

5.	Liu YH, Chen MT, He YY, Chen M, Liang JR, Jia FJ, et al. Cognitive impairment and depression precede increased HDL-C levels in middle-aged and older Chinese adults: cross-lagged panel analyses. Lipids in health and disease. 2024;23(1):288.<https://doi.org/10.1186/s12944-024-02285-9>.

6.	Sang N, Liu RC, Zhang MH, Lu ZX, Wu ZG, Zhang MY, et al. Changes in frailty and depressive symptoms among middle-aged and older Chinese people: a nationwide cohort study. BMC Public Health. 2024;24(1):301.<https://doi.org/10.1186/s12889-024-17824-3>.

7.	Zhang J, Jia X, Li Y, Li H, Yang Q. The longitudinal bidirectional association between sarcopenia and cognitive function in community-dwelling older adults: Findings from the China Health and Retirement Longitudinal Study. Journal of global health. 2023;13:04182.<https://doi.org/10.7189/jogh.13.04182>.

8.	Lu S, Wu YP, Mao ZF, Liang XH. Association of Formal and Informal Social Support With Health-Related Quality of Life Among Chinese Rural Elders. International Journal of Environmental Research and Public Health. 2020;17(4).<https://doi.org/10.3390/ijerph17041351>.

9.	Zhu W. The Impact of Social Support on the Mental Health of Cancer Patients: Evidence from China. Psycho-Oncologie. 2024;18(1):69-77.<https://doi.org/10.32604/po.2023.046593>.

10.	Guo L, Sang B, Li S, Xia Z, Li M, Yang M, et al. From starvation to depression: unveiling the link between the great famine and late-life depression. BMC Public Health. 2024;24(1):3096.<https://doi.org/10.1186/s12889-024-20604-8>.

11.	An S, Ouyang W, Wang S, Yuan J, Zhen X. Marital transitions and frailty among middle-aged and older adults in China: The roles of social support. SSM - Population Health. 2023;24:101497.<https://doi.org/10.1016/j.ssmph.2023.101497>.

12.	Jiang Z, Liu H, Deng J, Ye Y, Li D. Influence of intergenerational support on the mental health of older people in China. PloS one. 2024;19(4):e0299986.<https://doi.org/10.1371/journal.pone.0299986>.

13.	Wang P, Tao C. The influence of intergenerational support on cognitive functioning among middle-aged Chinese: Moderation by personal income. Archives of Gerontology and Geriatrics. 2024;122:105393.<https://doi.org/https://doi.org/10.1016/j.archger.2024.105393>.

14.	Hong Y, Xu W, Zhao L. The impact of grandchild care on depressive symptoms of grandparents in China: The mediating effects of generational support from children. Front Public Health. 2023;11:1043969.<https://doi.org/10.3389/fpubh.2023.1043969>.

15.	Lei X, Sun X, Strauss J, Zhang P, Zhao Y. Depressive symptoms and SES among the mid-aged and elderly in China: evidence from the China Health and Retirement Longitudinal Study national baseline. Social Science & Medicine. 2014;120:224-232.<https://doi.org/10.1016/j.socscimed.2014.09.028>.

16.	Zhang C, Lei X, Strauss J, Zhao Y. Health Insurance and Health Care among the Mid-Aged and Older Chinese: Evidence from the National Baseline Survey of CHARLS. Health Econ. 2017;26(4):431-449.<https://doi.org/10.1002/hec.3322>.
